# Genome-wide association studies and whole-genome prediction reveal the genetic architecture of KRN in maize

**DOI:** 10.1186/s12870-020-02676-x

**Published:** 2020-10-27

**Authors:** Yixin An, Lin Chen, Yong-Xiang Li, Chunhui Li, Yunsu Shi, Dengfeng Zhang, Yu Li, Tianyu Wang

**Affiliations:** Institute of Crop Sciences, Chinese Academy of Agricultural Sciences, Beijing, 100081 China

**Keywords:** Maize, Kernel row number, Genome-wide association study, Quantitative trait nucleotide, Whole-genome prediction

## Abstract

**Background:**

Kernel row number (KRN) is an important trait for the domestication and improvement of maize. Exploring the genetic basis of KRN has great research significance and can provide valuable information for molecular assisted selection.

**Results:**

In this study, one single-locus method (MLM) and six multilocus methods (mrMLM, FASTmrMLM, FASTmrEMMA, pLARmEB, pKWmEB and ISIS EM-BLASSO) of genome-wide association studies (GWASs) were used to identify significant quantitative trait nucleotides (QTNs) for KRN in an association panel including 639 maize inbred lines that were genotyped by the MaizeSNP50 BeadChip. In three phenotyping environments and with best linear unbiased prediction (BLUP) values, the seven GWAS methods revealed different numbers of KRN-associated QTNs, ranging from 11 to 177. Based on these results, seven important regions for KRN located on chromosomes 1, 2, 3, 5, 9, and 10 were identified by at least three methods and in at least two environments. Moreover, 49 genes from the seven regions were expressed in different maize tissues. Among the 49 genes, *ARF29* (Zm00001d026540, encoding auxin response factor 29) and *CKO4* (Zm00001d043293, encoding cytokinin oxidase protein) were significantly related to KRN, based on expression analysis and candidate gene association mapping. Whole-genome prediction (WGP) of KRN was also performed, and we found that the KRN-associated tagSNPs achieved a high prediction accuracy. The best strategy was to integrate all of the KRN-associated tagSNPs identified by all GWAS models.

**Conclusions:**

These results aid in our understanding of the genetic architecture of KRN and provide useful information for genomic selection for KRN in maize breeding.

## Background

Maize (*Zea mays* L.) arose from a single domestication event from its wild progenitor, teosinte, in southern Mexico approximately 9000 years ago and is now one of the most important cereal crops worldwide [1]. During domestication, its morphological characteristics, especially inflorescence architectures, differed profoundly [2, 3]. The shift from small ears in teosinte to larger ears in modern maize was accompanied by a dramatic increase in kernel row number (KRN) [4]. Thus, constant efforts have been made to explore the genetic basis underlying the striking diversities in inflorescence architecture and KRN in maize.

KRN is an important ear trait and is formed by multiple meristem types during female inflorescence development, including inflorescence meristems (IMs), spikelet pair meristems (SPMs), spikelet meristems (SMs) and floral meristems (FMs) [5]. To date, some genes have been cloned and found to be involved in complex regulatory networks responsible for meristem development and KRN modification by studying mutants [6–10]. However, these classical mutants show negative pleiotropy for other traits related to plant architecture and are difficult to directly use in maize breeding [11]. Therefore, linkage mapping and association mapping have been performed in naturally varying populations with the aim of identifying more elite natural alleles controlling KRN.

Although many quantitative trait loci (QTLs) related to KRN were identified by linkage mapping in biparental segregating populations, few have been successfully cloned due to their small genetic effects, except for *KRN4* [12] and *KRN1* [13]. Genome-wide association studies (GWASs) of KRN have also been conducted and revealed many quantitative trait nucleotides (QTNs) [14–16]. At the same time, GWAS results can be easily influenced by population structure and rare variants in natural populations [17]. Therefore, many statistical models have been developed to improve power for identifying genotype-phenotype associations when using the GWAS approach, such as the single-locus mixed linear model (MLM) method [18, 19] and the multilocus methods mrMLM [20], ISIS EM-BLASSO [21], pLARmEB [22], FASTmrEMMA [23], pKWmEB [24], and FASTmrMLM [25]. The MLM method is a single-locus fixed-single nucleotide polymorphism (SNP)-effect approach used in the case of a polygenic background to control population structure [18, 19]. To reduce the false positive rate (FPR), stringent Bonferroni correction is used for multiple testing correction in the MLM approach [26]. The multilocus method is an alternative GWAS procedure that is based on a random-SNP-effect model, and no multiple testing correction is needed [26]. There are two steps in this model. First, a reduced number of SNPs is selected through different algorithms, and the SNPs are then used in the multilocus model to detect true signals [20–26]. Recently, a few studies have implemented the above GWAS methods to detect important loci controlling different traits in rice [27], maize [28], flax [29], bread wheat [30] and upland cotton [31, 32].

Previous studies have revealed that KRN is quantitatively inherited and that the effects of a single genetic locus are generally small, which poses challenges for genetic improvement in maize breeding. Therefore, the best approach is to improve the ability to predict KRN by integrated analysis of more markers distributed throughout the whole genome. Genomic selection (GS), or whole-genome prediction (WGP), has the capacity to use full-genome data to increase breeding efficiency [33]. In previous studies, WGPs of KRN were performed in F_1_ hybrids between recombinant inbred lines [34], interconnected biparental maize populations [35] and 339 maize inbred lines [36], all of which showed that KRN was a trait suitable for genome-wide prediction. Liu et al. [15] showed that approximately 300 top KRN-associated tagSNPs were sufficient for predicting the KRN of inbred lines and hybrids using ridge regression best linear unbiased prediction (rr-BLUP). Based on these analyses, we are faced with determining how to select fewer markers to accurately predict KRN. Several studies reported that selecting association markers from the results of GWASs and including them as fixed effects in WGP models resulted in better performance than that achieved with single WGP models [37–39]. This might provide a way to simultaneously model different aspects of genetic architecture and is especially accessible to breeders [39].

In this study, we performed a GWAS of an association panel including 639 maize inbred lines based on the MaizeSNP50 BeadChip by using one single-locus method, the MLM method, and six multilocus methods, mrMLM, FASTmrMLM, FASTmrEMMA, pLARmEB, pKWmEB and ISIS EM-BLASSO. The common significant QTNs codetected by different methods and across different environments were analyzed, and the candidate genes related to KRN were further predicted. WGP was also performed using various KRN-related tagSNPs to dissect the genetic architecture of KRN.

## Results

### Natural variation in KRN within the association panel

**Fig. 1:**
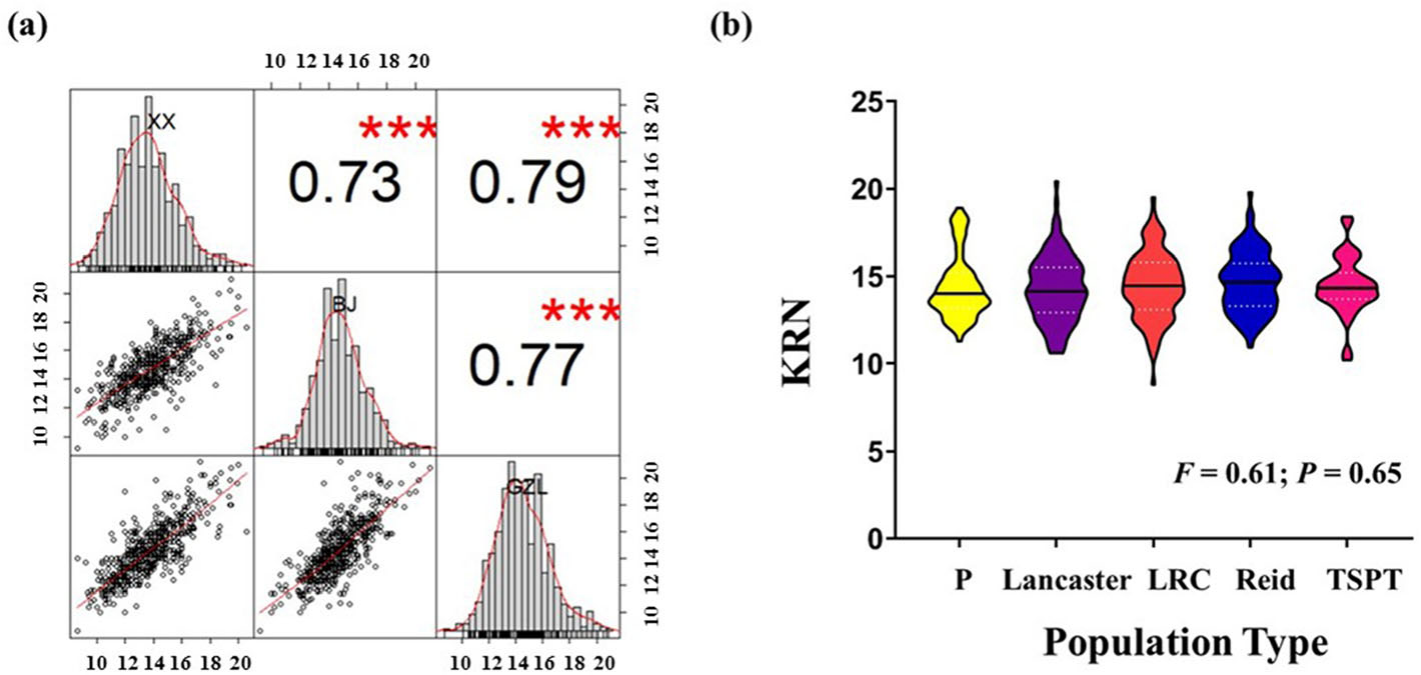
Phenotypic analysis. **a** Correlation analysis of the KRN phenotype among XX, BJ and GZL. The frequency distribution diagrams of KRN in three environments were plotted, and the correlation coefficient between each pair of environments was calculated. **b** Violin plots of KRN in the subgroups (P, Lancaster, TSPT, LRC, and Reid) of this association mapping panel

KRN was measured within our association panel, which included 639 maize inbred lines, in XX (Xinxiang in Henan Province, 35.19°N, 113.53°E), BJ (Beijing, 39.48°N, 116.28°E) and GZL (Gongzhuling in Jilin Province, 43.50°N, 124.82°E) in 2011 (Table S1). The results showed that KRN was normally distributed in each environment, and the KRNs among environments were highly positively correlated, with correlations ranging from 0.73 between XX and BJ to 0.79 between XX and GZL (Fig. 1a). KRN exhibited high broad-sense heritability (*H*^*2*^ = 0.90, Table 1), which was similar to the results of previous studies [14, 16]. Comparing KRN among the different environments, we found that it showed the smallest average (13.69), minimum (8.60) and maximum (20.60) values in XX, where all accessions were planted in summer (June). With increasing latitude, where the accessions were planted in spring (May), the average KRN increased (14.65 in BJ and 14.59 in GZL). The largest range (max - min) in KRN appeared in GZL (12.60), which had the longest day length (Table 1). Based on previous results [40], our association panel could be divided into five subgroups: Reid, tangsipingtou (TSPT), lvdahonggu (LRC), Lancaster and P. The KRN statistical analysis results of various subgroups are shown in Table S2. There were no significant differences in KRN among the five subgroups (Fig. 1b). These results indicated that KRN was a quantitative trait and that the phenotypic variation among the tested inbred lines in the association panel was beneficial for dissecting the genetic architecture of KRN.

**Table 1:** Phenotypic variance in KRN for 639 maize inbred lines in three environments

Env	Mean	Min	Max	***SD***	***CV*** (%)	***H***^***2***^
XX	13.69	8.60	20.60	2.02	14.76	0.90
BJ	14.65	9.20	21.00	1.69	11.56	
GZL	14.59	8.60	21.20	2.00	13.69	
BLUP	14.31	9.17	20.01	1.61	11.27	

*Env* environment, *XX* Xinxiang, *BJ* Beijing, *GZL* Gongzhuling, *Max* maximum, *Min* minimum, *SD* standard deviation, *CV* coefficient of variation, *H*^*2*^ broad-sense heritability

### QTNs for KRN identified by different methods

#### Single-locus analysis of KRN (MLM)

**Fig. 2:**
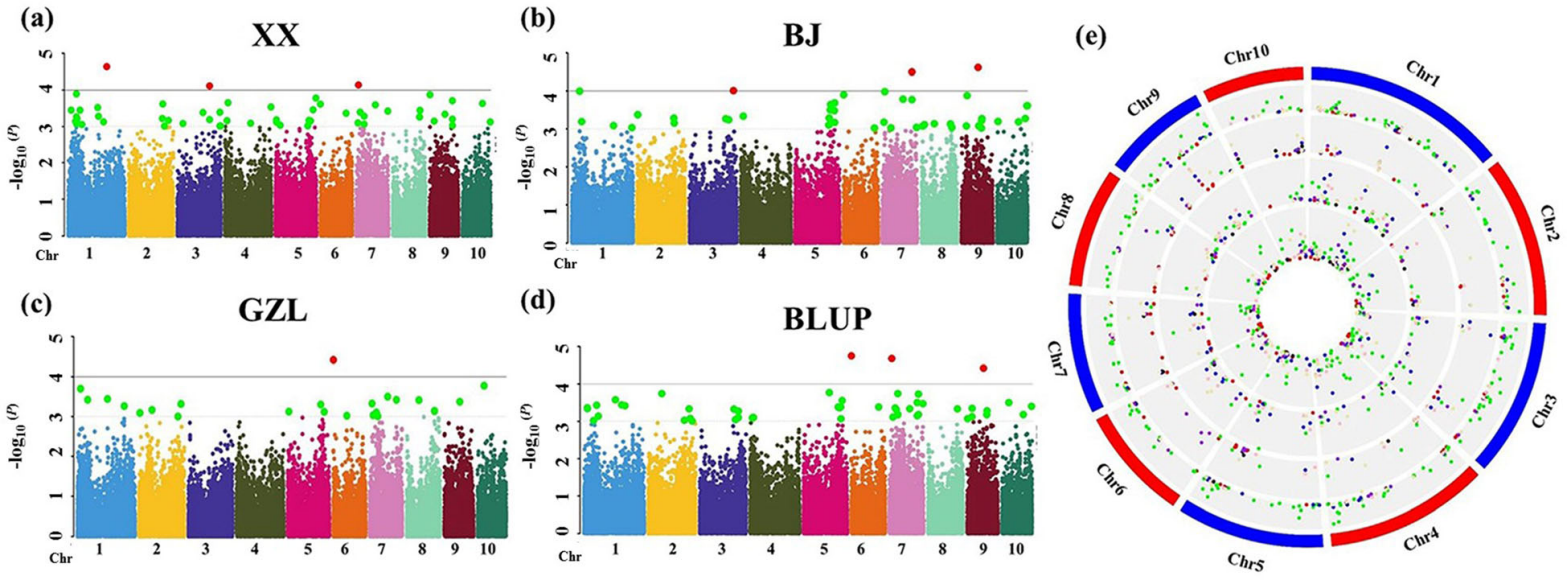
Genome-wide distribution of significant QTNs detected by different models under four conditions. **a** XinXiang (XX), Henan Province by the MLM method; **b** Beijing (BJ) by the MLM method; **c** Gongzhuling (GZL), Jilin Province, by the MLM method; **d** BLUP across the three environments by the MLM method; **e** The genome-wide distribution of all the significant QTNs identified by seven methods: the four circles from outside to inside show the distribution of significant QTNs identified in XX, BJ, and GZL and with BLUP, respectively. Dots of different colors represent QTNs mined by different GWAS models: red dots, MLM; green dots, mrMLM; blue dots, FASTmrMLM; black dots, FASTmrEMMA; pink dots, pLARmEB; purple dots, pKWmEB; pale goldenrod dots, ISIS EM-BLASSO

Based on the MaizeSNP50 BeadChip, we obtained 42,667 high-quality SNPs distributed on 10 maize chromosomes. Under the *P* < 0.0001 and *P* < 0.001 thresholds, 3/56, 3/46, 1/24, and 3/51 KRN-associated QTNs were found in XX (Fig. 2a), in BJ (Fig. 2b), in GZL (Fig. 2c) and with BLUP (Fig. 2d), respectively. To account for overcorrection in this model, the *P* < 0.001 threshold was selected to identify KRN-associated QTNs. Finally, 177 QTNs were found to be associated with KRN, and the proportion of phenotypic variance explained (PVE) by these individual QTNs ranged from 1.84 to 4.01% (Table S3).

#### Multiple-locus analysis of KRN

Using different multiple-locus models, we identified different numbers of significant QTNs for KRN in XX, BJ, and GZL and together with BLUP across all locations. These QTNs were unevenly distributed on 10 chromosomes, with the most QTNs on Chr. 1 and the fewest on Chr. 8 (Fig. 2e). Specifically, 15 (FASTmrEMMA)-177 (mrMLM) QTNs in XX, 11 (FASTmrEMMA)-30 (ISIS EM-BLASSO) QTNs in BJ, 12 (FASTmrEMMA)-55 (mrMLM) QTNs in GZL and 11 (FASTmrEMMA)-106 (mrMLM) QTNs for BLUP were identified by the six different methods (Table S4). Comparative analysis of the GWAS results among different statistical approaches showed that FASTmrEMMA detected the fewest QTNs in all the environments, while mrMLM detected the most QTNs in all the environments, except for BJ (Table S4). QTN overlap analysis among the seven methods indicated that the common QTNs codetected by at least two methods accounted for more than 40% of the QTNs in different environments (Figure S1a and Table S5, 42% in XX, 62% in BJ, 58% in GZL and 47% with BLUP). For example, 65 common QTNs representing 30 loci were codetected by two methods in XX, and 39 common QTNs representing 13 loci, 28 common QTNs representing 7 loci, 25 common QTNs representing 5 loci, and 6 common QTNs representing 1 locus were codetected by three, four, five and six methods, respectively (Figure S1a and Table S5). No QTNs were identified by all 7 methods in different locations. Overall, ISIS EM-BLASSO, which detected the third largest number of QTNs, identified the most codetected QTNs, followed by FASTmrMLM (Figure S1a and Table S5). Comparative analysis of the GWAS results among the different environments showed that the majority of the QTNs identified by the MLM method and ISIS EM-BLASSO were repeatedly detected in different locations (Figure S1b, Table S6).

Overall, comparing our GWAS results with those of previous studies, we found that some important genes controlling inflorescence architecture in maize were located within 200 kb of the significant QTNs, including *CT2* (Zm00001d027886), *FEA3* (Zm00001d040130), *BAD1* (Zm00001d005737), *RA1* (Zm00001d020430), and *VT2* (Zm00001d008700) (Table S13).

### Annotation and expression of candidate genes for KRN

**Fig. 3:**
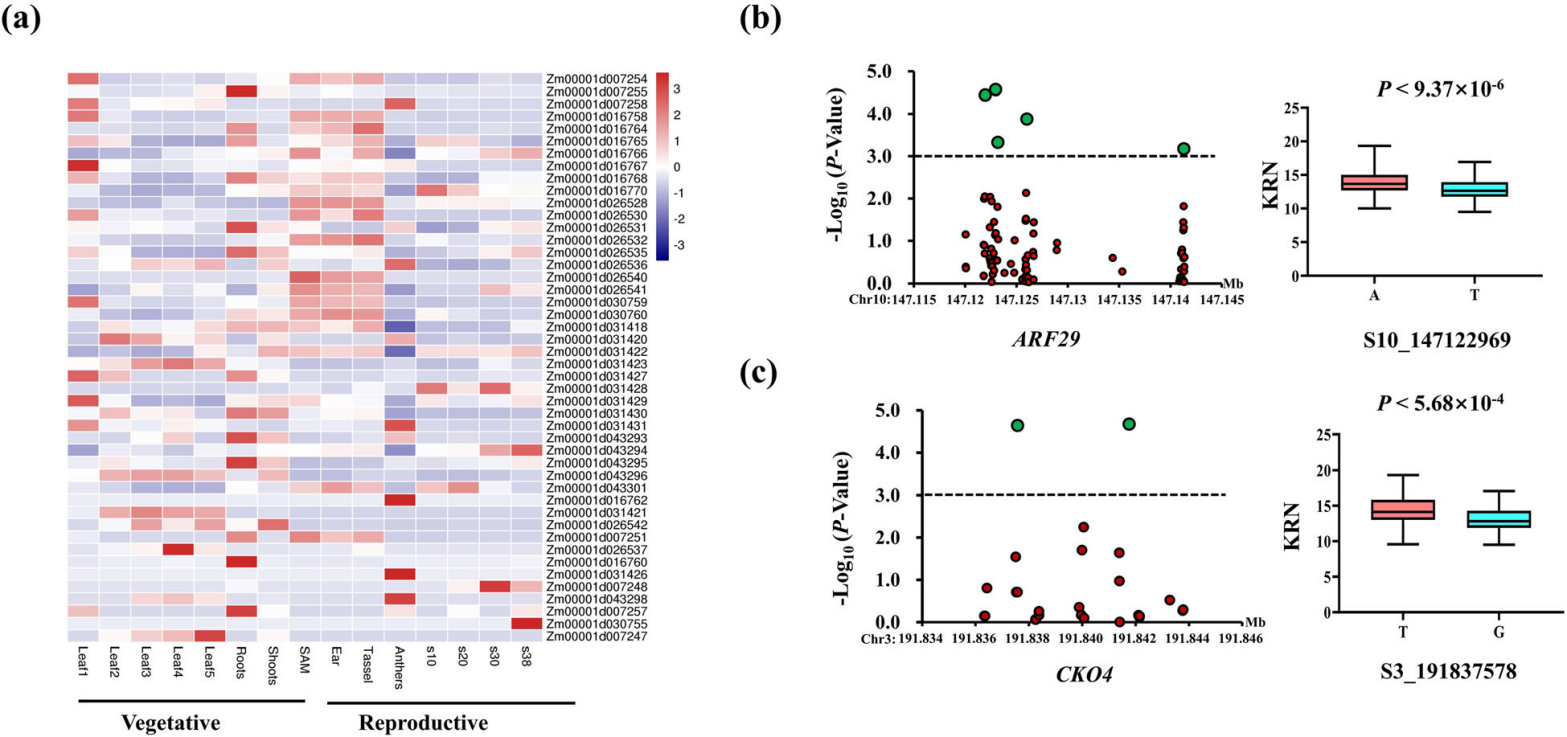
Candidate gene analysis of KRN. **a** Expression heatmap of the genes located in the codetected regions. All expression data were collected from inbred B73. Leaf 1 means the leaf base; leaf 2 means a 1-cm leaf; leaf 3 means a 4-cm leaf; leaf 4 means the leaf tip; leaf 5 means the leaf at 20 days after pollination (DAP); S10 means the kernel at 10 DAP. **b** *ARF29* (Zm00001d026540) gene association mapping using the Ames 228 panel. **c** *CKO4* (Zm00001d043293) gene association mapping

To obtain reliable significant QTNs and predict the candidate genes for KRN, only the QTNs simultaneously identified by at least three methods (either single-locus or multilocus) and in at least two environments were used for the next analysis. Finally, seven QTNs controlling KRN were obtained (Table 2). The seven QTNs were located on chromosomes 1, 2, 3, 5, 9, and 10, and the PVE by these QTNs ranged from 1.06 to 5.21%. Based on the linkage disequilibrium (LD) in the association panel (Figure S2), 49 genes around the QTNs (200 kb upstream and downstream) were obtained, and their expression varied widely in different maize tissues (Fig. 3a and Table S7). For example, Zm00001d016760, which encodes the abscisic acid stress ripening 6 protein, is highly expressed in the roots, and Zm00001d031426, which encodes serine/threonine-protein kinase, and Zm00001d043298, which encodes a P-loop containing nucleoside triphosphate hydrolase superfamily protein, are highly expressed in tassels and anthers. Among the 49 genes, 22 were differentially expressed in different spike development mutants (Table S8); i.e., the *ra1*, *ra2* and *ra3* mutants had abnormal highly branched tassels and ears, with the ears displaying a very large KRN [41]; the *kn1* mutant had smaller ears and fewer spikelets [42]. This result suggested that these 22 genes might be involved in ear development in maize.

**Table 2:** Significant KRN-associated QTNs codetected in at least two environments and by at least three models

SNP	Chr	Pos	Single-locus GWAS (MLM)		Multilocus GWAS		
			LOD	PVE (%)	LOD	PVE (%)	Methods^**1**^
PZE-101124566	1	156,580,056	3.44	3.00	4.60–11.63	1.91–3.02	2, 3, 4, 5, 6, 7
PZE-101144585	1	187,526,525	3.13	2.00	4.39–5.95	1.84–3.51	3, 4, 5, 7
PZE-102176259	2	219,023,013	3.32	3.00	3.41–4.17	1.06–2.04	2, 3, 4, 7
PUT-163a-110,967,306-138	3	191,981,941	3.28	2.56	8.17–11.77	1.62–3.37	2, 5, 6
PZE-105114980	5	171,187,130	/	/	4.35–8.20	1.15–2.29	2, 3, 5,6,7
PZE-109047930	9	79,941,271	4.61	4.00	5.73–10.40	2.43–5.21	2, 3, 5, 6, 7
PZE-110106563	10	146,944,098	3.61	3.00	3.65–5.25	1.18–2.38	2, 3, 4, 5, 6, 7

Methods^1^: Numbers 1 to 7 represent different GWAS methods: 1: MLM; 2: mrMLM; 3: FASTmrMLM; 4: FASTmrEMMA; 5: pLARmEB; 6: pKWmEB; 7: ISIS EM-BLASSO

Interestingly, we found that Zm00001d026540 (encoding auxin response factor 29, ARF29), which was located within 200 kb downstream of PZE-110106563 on Chr. 10 and was detected by the MLM method and all six multilocus GWAS methods (Table 2), had higher expression in SAMs and ears than in other tissues (Table S7). Candidate gene association mapping was also performed. The SNPs within *ARF29* and the 10-kb promoter and 10-kb region downstream of *ARF29* were obtained from maize HapMap3 [43]. The KRN of 282 inbred lines was measured in six environments (see Methods), and the BLUP values were calculated. The MLM mapping result showed that five SNPs (two SNPs in the gene and three SNPs in the region upstream of the gene) around *ARF29* were significantly related to KRN (Fig. 3b and Table 3). ARF29 can bind the *Bif1* (which is related to SAM development and final KRN) promoter by recognizing the TTTCGG motif [44, 45]. The S10_147,122,969 SNP, located within the gene body, was significantly associated with KRN. Two alleles for this SNP (A/T) were present in this panel, with the A allele conferring a higher KRN. Cytokinins also play an important role in the development of immature spikes and the formation of final KRN [46]. For example, *UB3* regulates KRN by the cytokinin pathway and CLAVATA-WUSCHEL pathway [46]. In this study, *CKO4* (Zm00001d043293, encoding cytokinin oxidase protein) was detected as being located within 200 kb upstream of PUT-163a-110,967,306-138 on Chr. 3 by four GWAS methods (MLM, mrMLM, pLARmEB, and pKWmEB, Table 2), and candidate gene association mapping of *CKO4* was also conducted. The SNPs and KRN were also obtained from HapMap3 and 282 inbred lines. The MLM results showed that two SNPs located upstream of *CKO4* were significantly associated with KRN (Fig. 3c and Table 3). The S3_191,837,578 SNP had two alleles (T/G), and the T allele was associated with a higher KRN but had a lower frequency. Therefore, this allele may not be widely useful in maize breeding.

**Table 3:** Candidate gene association analysis

Gene ID	SNP^1^	Chr	Pos	LOD	PVE	Allele	Frequency
*ARF29*	S10_147,122,969	10	147,122,969	4.57	8.97%	A/T	127/99
	S10_147,121,954	10	147,121,954	4.44	8.98%	G/A	94/90
	S10_147,126,021	10	147,126,021	3.88	7.58%	T/A	161/27
	S10_147,123,193	10	147,123,193	3.33	5.30%	A/C	119/110
	S10_147,141,311	10	147,141,311	3.17	4.92%	C/G	211/21
*CKO4*	S3_191,837,578	3	191,837,578	4.64	7.85%	G/T	177/45
	S3_191,841,761	3	191,841,761	4.67	6.99%	T/G	236/16

^1^ The significant SNPs calculated by the MLM method in regional association mapping based on the 282 inbred lines

### Whole-genomic prediction of KRN

**Fig. 4:**
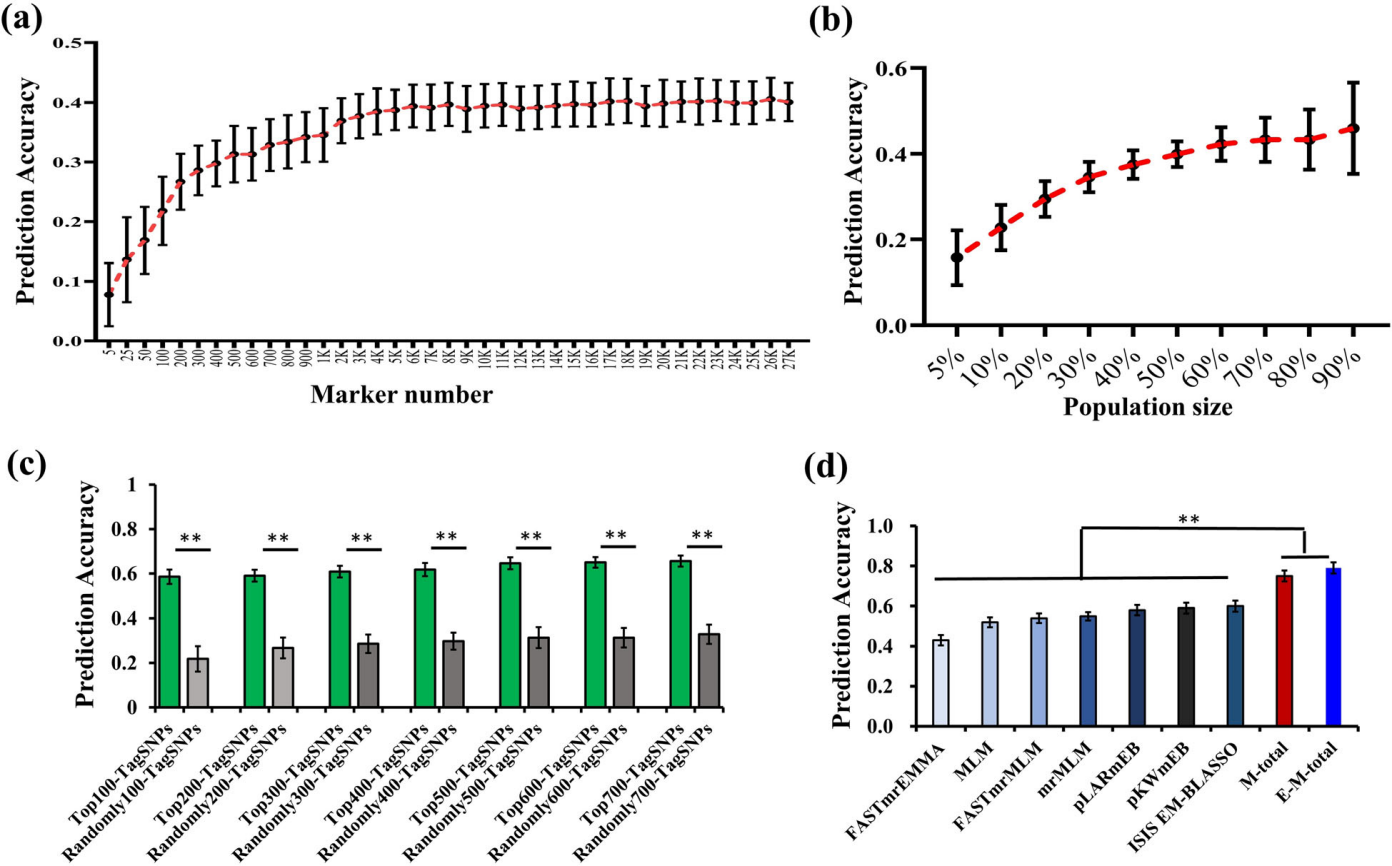
Whole-genome prediction of KRN in the inbred lines. **a** The KRN prediction accuracy for different numbers of randomly selected tagSNPs (from 5 to 27,000) based on BLUP values by using the rrBLUP model. **b** KRN prediction accuracy for different training population sizes. **c** Comparison of prediction accuracy between the top tagSNPs and random tagSNPs. **, *P* < 0.01. **d** Comparison of the prediction accuracy of different tagSNPs identified by different models. **, *P* < 0.01

We first analyzed the LD blocks of all markers using the threshold value *r*^*2*^ > 0.2 and obtained 27,688 tagSNPs in our association panel. Then, we randomly selected different numbers of tagSNPs, from 5 to 27,000, in the whole genome to calculate the prediction accuracies for KRN of the inbred lines, which was calculated as a correlation between predicted and true values from the simulations. The results showed that the prediction accuracies increased as the number of tagSNPs increased (Fig. 4a and Table S9). More specifically, the prediction accuracies sharply increased when the number of tagSNPs increased from 5 to 500 and then slowly increased when the number of tagSNPs increased from 400 to 2000. Once the number exceeded 2000, the prediction accuracies maintained a consistently high level. Although a large number of tagSNPs were used to predict KRN, the prediction accuracies were still less than 0.5. The effects of training population size on the prediction accuracy were also assessed based on a marker number of 14,000 (approximately 50% of the total tagSNPs). In the association panel, the prediction accuracies improved with increasing training population size. When the training population size increased from 50 to 90%, a slight increase in prediction accuracy was observed (Fig. 4b and Table S10).

To better understand the genetic architecture of KRN and improve the ability to predict it, we ranked the 27,688 tagSNPs according to their significance in relation to KRN, as obtained by the MLM method, to obtain the top tagSNPs. We found that these top tagSNPs had a higher prediction accuracy (ranging from 0.58 for the top 100 tagSNPs to 0.66 for the top 700 tagSNPs) than randomly selected tagSNPs (ranging from 0.22 for 100 random tagSNPs to 0.33 for 700 random tagSNPs) (Fig. 4c and Table S11).

The tagSNPs representing the significant QTNs detected by different models based on BLUP were collected and used to calculate prediction accuracies for KRN in our association panel. The results showed that these tagSNPs identified by different methods had different prediction accuracies ranging from 0.43 (FASTmrEMMA) to 0.60 (ISIS EM-BLASSO) (Fig. 4d and Table S12). We also found that the tagSNPs associated with KRN identified by the same method showed different prediction accuracies in diverse environments (Figure S3 and Table S12). To explore whether using the codetected QTNs in different GWAS methods could increase prediction accuracies for KRN, we selected the common QTNs identified by at least two, three, four, five or six methods to obtain the predictions. The results showed that only the common QTNs identified by at least two methods (common ≥2) could maintain predictability at a high level; other common QTNs had no advantage in predicting KRN, which may be due to the smaller QTN numbers (Figure S3 and Table S12).

Additionally, to improve the prediction ability, we put the KRN-related tagSNPs detected by seven methods together in a single environment (204 in XX, 87 in BJ, 118 in GZL and 167 for BLUP), namely, M-total tagSNPs, to conduct KRN prediction. As a result, we found that the prediction accuracies were improved sharply and reached 0.74 in XX, 0.66 in BJ, 0.75 in GZL and 0.75 for BLUP (Fig. 4d and Table S12). These predictabilities were much higher than those of the single method in each environment (Table S12). Then, we collected the tagSNPs associated with KRN from all methods and all environments, namely, E-M-total tagSNPs, and obtained 439 tagSNPs in total. However, there was only a slight increase in prediction accuracy (ranging from 0.68 in BJ to 0.79 for BLUP for the 439 tagSNPs) when we used the much higher number of E-M-total tagSNPs compared to the fewer M-total tagSNPs (Fig. 4d and Table S12).

## Discussion

To date, the GWAS approach has been widely used to investigate the genetic basis of important traits in many species by calculating the association between genotypic and corresponding phenotypic variations [47]. To identify true association signals, many statistical methods based on different algorithms have been established. In this study, we selected one single-locus method, MLM, and six multilocus methods, mrMLM, FASTmrMLM, FASTmrEMMA, pLARmEB, pKWmEB and ISIS EM-BLASSO, to perform comprehensive GWAS mapping of KRN in our association panel. Among the seven methods, mrMLM identified the largest number of QTNs, FASTmrEMMA identified the fewest QTNs, and ISIS EM-BLASSO identified the most codetected QTNs, which were consistent with the results reported by Cui et al. [27] for salt-tolerance loci in rice. Therefore, multilocus models are valuable alternative methods for GWASs of KRN in maize. Additionally, a small number of common QTNs codetected by different methods was also observed in the study of Peng et al. [30] for free amino acid levels in bread wheat.

Comparing our GWAS results with those of previous studies, we found that some important genes controlling inflorescence architecture in maize were located within 200 kb of significant QTNs (Table S13), including *CT2* (Zm00001d027886), *FEA3* (Zm00001d040130), *BAD1* (Zm00001d005737), *RA1* (Zm00001d020430), and *VT2* (Zm00001d008700). Among these genes, *CT2* [7] and *FEA3* [10] function in CLAVATA-WUSCHEL feedback signaling, and their mutations result in enlarged and fasciated ear primordia and increased KRN. *BAD1* [48] and *RA1* [41], both of which encode transcription factors, are involved in the genetic regulation of the floral branch system by the ROMASO pathway in maize. *VT2* [49] functions in auxin biosynthesis and has dramatic effects on vegetative and reproductive development, and mutant ears show obvious defects. Additionally, approximately 60% of the significant QTNs within LD regions were codetected by previous GWAS mapping of inflorescence development, and some of these loci were pleiotropic [14, 15].

WGP is also an effective method in animal breeding and plant improvement [50]. Because KRN is mainly controlled by additive loci, we selected the rrBLUP additive model to conduct WGP [51]. As expected, prediction accuracy increased as the number of randomly selected tagSNPs increased, which was consistent with the finding of Liu et al. [15] and determined by the influence of marker density on WGP [50]. However, the randomly selected tagSNPs showed a low predictive ability, and thus, we decided to combine the GWAS results with WGP to explore the best marker dataset for KRN prediction. As a result, higher prediction levels were easily reached when using the significant tagSNPs, and the moderate to high values were consistent with those reported by Liu et al. [15], Guo et al. [34], Riedelsheimer et al. [35] and Xu et al. [36]. This result suggested that integrating significant signals from GWASs into WGP models as fixed effects was effective for enhancing the prediction of KRN. A similar conclusion was reached by Liu et al. [15] for KRN, by Bian and Holland [52] for resistance to southern leaf blight (SLB) and gray leaf spot (GLS) and plant height (PHT) in maize and by Spindel et al. [39] for tropical rice improvement. Although different evaluations of WGP models incorporating peak GWAS signals have been performed in maize and sorghum [53], our research indicated that the use of QTNs passing a certain threshold in the above GWAS methods as fixed effects in the rrBLUP model is a powerful tool for KRN prediction, which was a trait-specific consideration in the given population in this study.

Based on the results of this study, we suggest that KRN is controlled by many additive loci and that the rrBLUP model can be used for KRN prediction in maize inbred lines. The combined utilization of different GWAS methods is helpful for predicting candidate genes and KRN in maize breeding.

## Conclusions

In this study, multiple GWAS methods were used to identify significant QTNs for KRN in maize. The seven GWAS methods revealed different numbers of KRN-associated QTNs, ranging from 11 to 177. Based on these results, seven important regions for KRN located on chromosomes 1, 2, 3, 5, 9, and 10 were identified by at least three methods and in at least two environments. Moreover, 49 genes from the seven regions were expressed in different maize tissues. Among the 49 genes, *ARF29* (Zm00001d026540, encoding auxin response factor 29) and *CKO4* (Zm00001d043293, encoding cytokinin oxidase protein) were significantly related to KRN, based on expression analysis and candidate gene association mapping. WGP of KRN was also performed, and we found that the KRN-associated tagSNPs achieved a high prediction accuracy. The best strategy was to integrate the total KRN-associated tagSNPs identified by all GWAS models. These results will facilitate our understanding of the genetic basis of KRN and provide important candidate genes for further research on this important trait.

## Methods

### Plant materials and phenotyping

An association panel of 639 maize inbred lines, representing a wide range of genetic diversity of temperate inbred lines in China [54], was collected for GWASs. We declare that all plant materials comply with the ‘Convention on the Trade in Endangered Species of Wild Fauna and Flora’ in this study. The plant materials used in this study were conserved in our lab.

All the accessions were planted following a randomized block design of three replicates in three environments in 2011: Gongzhuling in Jilin Province (43.50°N, 124.82°E), Xinxiang in Henan Province (35.19°N, 113.53°E) and Beijing (39.48°N, 116.28°E) in 2011. For descriptive purposes, the three environments were designated GZL, XX and BJ, respectively. At each location, the field experiments include in a single row 3 m in length, with 0.6 m between adjacent rows and 12 individual plants per row. The Institute of Crop Science of the Chinese Academy of Agricultural Sciences has established experimental field bases at all the above locations. The Institute of Crop Science approved the field experiments, and field management followed local maize management practices. In this study, the field studies did not involve endangered or protected species.

Five ears were harvested from each line, and KRN was evaluated in the middle part of the ears [54]. BLUP values were calculated using the SAS PROC MIXED model, with genotype, environment and replicate as random effects [14, 55]. The broad-sense heritability (*H*^*2*^) of KRN was calculated according to Wu et al. [40]. The coefficient of variation was calculated as *CV* (%) = SD/mean, where SD and mean refer to the standard deviation and mean, respectively, of KRN in each environment [55].

### DNA extraction and genotyping

Young leaves of five plants of each maize line according were collected for genomic DNA extraction. We extract the genomic DNA followed the cetyltrimethylammonium bromide (CTAB) method [56]. All samples were quality checked and genotyped using the MaizeSNP50 BeadChip, which is an Illumina BeadChip array of 56,110 maize SNPs developed from the B73 reference sequence [57]. Then, the successfully called SNPs with a missing rate of more than 20% and minor allele frequency (MAF) of < 0.05 were excluded from the genotyping dataset [58]. After that, 42,667 high-quality SNPs were used in further analysis.

### GWAS mapping

One single-locus method, MLM, and six multilocus methods, including mrMLM, FASTmrMLM, FASTmrEMMA, pLARmEB, pKWmEB, and ISIS EM-BLASSO, were used in this study. Alleles of each polymorphic locus with a minor frequency > 0.05 were used for further analysis. A kinship matrix was calculated and principal component analysis (PCA) was performed with the TASSEL 5.2 program [59]. An MLM controlling for population structure (Q) and kinship (K) (MLM Q + K) was also generated in TASSEL 5.2 [18, 19]. Six multilocus GWAS mapping methods were used along with the software package mrMLM.GUI v3.2 in the R environment (http://127.0.0.1:5846/) [26]. All parameters were set at default values, the critical threshold of significant associations for the MLM was set at –log_10_^*P*^ ≥ 3, and the logarithm of odds (LOD) score for the six multilocus methods was set at ≥3 [26].

### Candidate gene analysis

The LD decay with physical distance in our association panel was calculated in TASSEL 5.2 to be 200 kb (Figure S2). The candidate genes in the 200-kb region around significant QTNs detected by at least three models and in two environments were identified based on the B73 reference genome V4 from MaizeGDB (https://www.maizegdb.org/). Expression data for these genes were collected from previous studies [42, 60]. Genome fragments containing the SNPs within the selected genes, including the 10-kb promoter region, the gene bodies and the 10-kb region downstream of the genes, were obtained from the maize HapMap3 dataset [43]. The candidate gene mapping analyses were conducted on a global maize association mapping panel of 282 diverse lines. The phenotypes of this association panel were provided in our previous report [40], and KRN was measured in six environments, including Beijing, Xinxiang in Henan, and Urumqi in Xinjiang in 2009 and 2010. Association analysis was conducted by the MLM method in TASSEL 5.2, controlling for population structure (Q) and kinship (K). The first three principal components (PCs), which were analyzed in a previous study [40], were used as covariants to control for existing population structure in the 282-line association mapping panel. Significant marker-trait associations were declared at –log_10_^*P*^ > 3.

### Genomic prediction of KRN

To predict the KRN of the inbred lines, we estimated predictability by WGP. We grouped the LD blocks in PLINK software [61] using the threshold value *r*^*2*^ > 0.2 and identified tagSNPs according to the LD blocks. The ridge regression best linear unbiased prediction (rrBLUP) package was used to perform genomic prediction in R [62]. We randomly selected half of the lines of our association panel as the training population (320 inbred lines) and the remaining 319 inbred lines as the validation population [15]. We used the KRN-related tagSNPs identified by different methods to perform genomic prediction of KRN for the inbred lines under four conditions (XX, BJ, GZL and BLUP). Simultaneously, 5 to 27,000 randomly selected tagSNPs, the total tagSNPs related to KRN identified by the seven methods in a single environment (M-total tagSNPs), the total tagSNPs for KRN from all methods and environments (E-M-total tagSNPs) and the common tagSNPs for KRN detected by at least two, three, four, five, or six methods were also used for the same procedure. The random sampling of tagSNP numbers, the training and validation populations and the predictions were all repeated 100 times.

## Supplementary information

**Additional file 1: Figure S1.** Common QTNs codetected with different models and in different environments. a, The common QTNs codetected by different methods. The X-axis represents different environments. The Y-axis represents the corresponding number of significant QTNs detected by only one method and by at least two, three, four, five, six or seven methods. b, The common QTNs codetected across different locations.

**Additional file 2: Figure S2.** LD decay with physical distance in our association panel.

**Additional file 3: Figure S3.** Whole-genome prediction of KRN in the inbred lines. The bars with different colors represent prediction accuracies for the KRN when using tagSNPs identified by different models. *P*-values were estimated based on the two-tailed Student’s t-test. ^***^: *P*-value < 0.0001; NS: *P*-value > 0.05.

**Additional file 4: Table S1**. A list of material information in our association panel.

**Additional file 5: Table S2.** Descriptive statistics of KRN from the subgroups in the association panel.

**Additional file 6: Table S3.** The significant QTNs for KRN identified by the MLM method.

**Additional file 7: Table S4.** The significant QTNs for KRN identified by six multilocus methods.

**Additional file 8: Table S5**. The common QTNs codetected by different methods.

**Additional file 9: Table S6**. The common QTNs codetected across different locations.

**Additional file 10: Table S7**. The expression of the candidate genes in different maize tissues.

**Additional file 11: Table S8.** Genes related to spike mutation in maize.

**Additional file 12: Table S9**. KRN prediction accuracies for different numbers of randomly selected tagSNPs (from 5 to 27,000).

**Additional file 13: Table S10**. KRN prediction accuracies for different training population sizes.

**Additional file 14: Table S11**. Comparison of prediction accuracy between the top tagSNPs and random tagSNPs.

**Additional file 15: Table S12**. The prediction accuracies for KRN of the inbred lines obtained using the tagSNPs representing the significant QTNs identified by different methods.

**Additional file 16: Table S13**. Comparison of our GWAS results with QTNs detected in previous studies.

## Data Availability

The datasets used and/or analyzed during the current study are available from the corresponding author upon reasonable request.

## Abbreviations

BJ: Beijing
FMs: Floral meristems
GWAS: Genome-wide association study
GZL: Gongzhuling
IM: Inflorescence meristem
KRN: Kernel row number
LD: Linkage disequilibrium
MLM: Mixed linear model
QTL: Quantitative trait locus
QTN: Quantitative trait nucleotide
SMs: Spikelet meristems
SNP: Single nucleotide polymorphism
SPMs: Spikelet pair meristems
XX: Xinxiang
