# Supplementary figures and images for: Genome-wide association studies and whole-genome prediction reveal the genetic architecture of KRN in maize
Source: BMC Plant Biol. 2020 Oct 27;20:490. doi: 10.1186/s12870-020-02676-x (PMC7590725; doi:10.1186/s12870-020-02676-x)

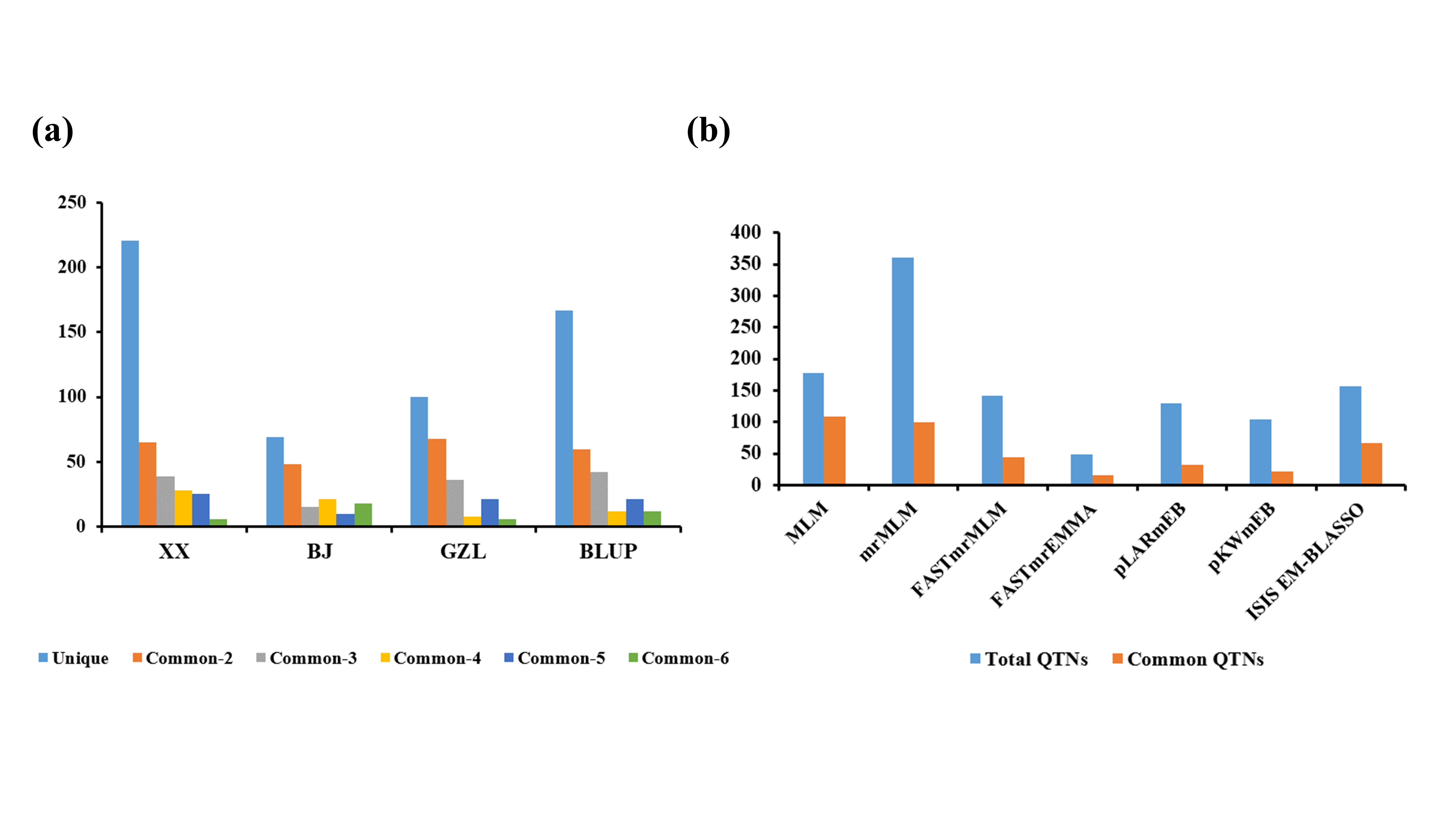

Supplement: Supplementary file 1 — Additional file 1: Figure S1. Common QTNs codetected with different models and in different environments. a, The common QTNs codetected by different methods. The X-axis represents different environments. The Y-axis represents the corresponding number of significant QTNs detected by only one method and by at least two, three, four, five, six or seven methods. b, The common QTNs codetected across different locations. [file 12870_2020_2676_MOESM1_ESM.tiff]

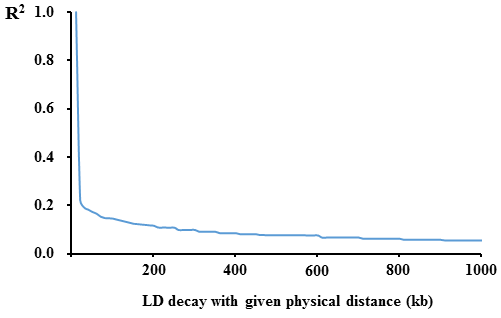

Supplement: Supplementary file 2 — Additional file 2: Figure S2. LD decay with physical distance in our association panel. [file 12870_2020_2676_MOESM2_ESM.tiff]

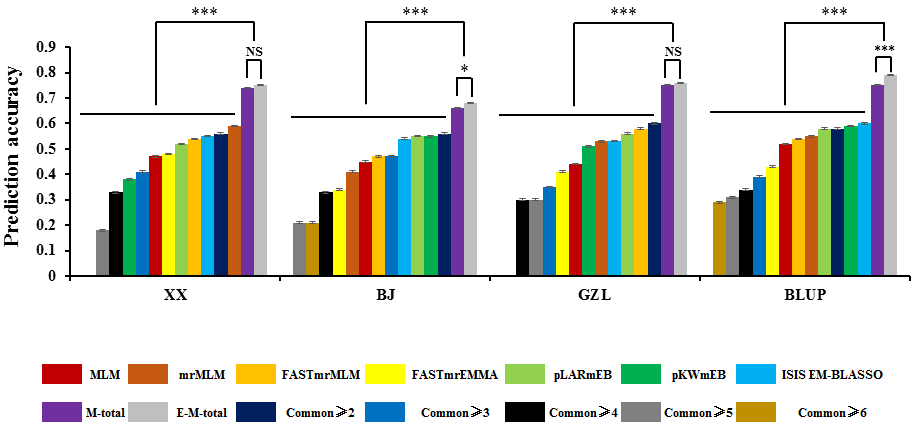

Supplement: Supplementary file 3 — Additional file 3: Figure S3. Whole-genome prediction of KRN in the inbred lines. The bars with different colors represent prediction accuracies for the KRN when using tagSNPs identified by different models. P-values were estimated based on the two-tailed Student’s t-test. ***: P-value < 0.0001; NS: P-value > 0.05. [file 12870_2020_2676_MOESM3_ESM.tiff]
